# Coexisting nutcracker phenomenon and superior mesenteric artery syndrome in a patient with IgA nephropathy: A case report

**DOI:** 10.1097/MD.0000000000026611

**Published:** 2021-07-16

**Authors:** Chenghua Wang, Fengmei Wang, Bing Zhao, Liang Xu, Bing Liu, Qi Guo, Xiaowei Yang, Rong Wang

**Affiliations:** a Department of Emergency center, Provincial Hospital affiliated to Shandong First Medical University, Jinan, Shandong, China; b Department of Nephrology, Zhong Da Hospital, Southeast University School of Medicine, Nanjing, Jiangsu, China; c Department of Nephrology, Shandong Provincial Hospital Affiliated to Shandong University, Jinan, Shandong, China.

**Keywords:** acute renal failure, case report, IgA nephropathy, nutcracker syndrome, nutcracker and superior mesenteric artery syndrome

## Abstract

**Rationale:**

Nutcracker and superior mesenteric artery (SMA) syndrome share the same pathogenesis, but the simultaneous occurrence of both diseases is quite rare. A combination of the nutcracker syndrome and IgA nephropathy has previously been reported. Herein, we report what we believe is the first case of coexisting nutcracker and SMA syndrome in a patient with IgA nephropathy.

**Patient concerns:**

A 15-year-old Chinese boy who was diagnosed with IgA nephropathy at 8 years of age presented with gross hematuria, fatigue, anorexia, nausea, and recurrent abdominal distension for 1 week without any obvious evidence of preceding infection. Laboratory data showed macroscopic hematuria, heavy proteinuria, and relatively normal renal function. Doppler ultrasonography and upper gastrointestinal gastrografin study were performed, respectively. Since his renal function deteriorated after admission, repeated renal biopsy was performed.

**Diagnoses:**

IgA nephropathy with nutcracker phenomenon and SMA syndrome.

**Intervention:**

Immunosuppressive therapy combined with conservative therapy for superior mesenteric artery syndrome.

**Outcomes:**

One month later, his abdomen symptoms such as anorexia and abdominal distension eased a lot with body weight increase of about 3 kg. After 6 months of follow-up, his body weight increased to 57 kg, serum creatinine decreased to 63 μmol/L, and urine microscopy showed 75.5 RBC/high-power field with 0.3 g urine protein per day.

**Lessons:**

Although the association between vascular compression and IgA nephropathy (IgAN) has not been elucidated yet, combination of nutcracker syndrome and SMA syndrome should be considered in patients with IgAN. The combination may increase the complexity of the disease, and renal biopsy should not be hesitated for differential diagnosis.

## 1 Introduction

IgA nephropathy (IgAN) is the most common chronic glomerular disease globally, especially in Pacific Asian regions. Intermittent gross hematuria and persistent asymptomatic microscopic hematuria with or without mild to moderate proteinuria are the 2 major clinical presentations of IgAN.

The nutcracker phenomenon refers to the entrapment of left renal vein (LRV) most often between abdominal aorta and superior mesenteric artery (SMA) with a rare type of compression between abdominal aorta and vertebral column.^[1,2]^ LRV entrapment is also a documented cause of hematuria and proteinuria.^[3]^ Some researchers have proposed that nutcracker should be defined only when the clinical symptoms are present along with compatible radiologic findings,^[4]^ because asymptomatic dilatation of LRV frequently shown on ultrasound or computed tomography has been regarded as a finding of a normal variant.^[5]^ However, LRV entrapment has been reported to coexist with some idiopathic glomerular disease in some case reports, among which coexistence with IgAN were the most commonly reported ones.^[6–11]^ The combination increases the difficulty of differentiating the primary cause of renal manifestations and influences the choice of therapy.

Less often, the third portion of the duodenum courses in front of the LRV between the aorta and the SMA. If the duodenum is compressed by the SMA due to a reduced aortomesenteric angle, upper intestinal obstruction occurs, known as the SMA syndrome (Wilkie syndrome). Nutcracker and SMA syndrome have the same pathogenesis, but the simultaneous occurrence of both diseases is quite rare.^[12]^ This is a report on a case of concurrent nutcracker and SMA syndrome in a 15-year-old Chinese male juvenile who has been diagnosed with IgA nephropathy for 8 years. To our knowledge, this is the first reported case of coexisting nutcracker, SMA syndrome and IgA nephropathy.

## 2 Case report

A 15-year-old boy was admitted to our hospital complaining of gross hematuria with fatigue, anorexia, nausea, and recurrent abdominal distension for 1 week without any obvious evidence of preceding infection. He had been diagnosed with IgAN at 8 years of age after presenting with gross hematuria with a baseline serum creatinine of 60 μmol/L. His renal biopsy at the time showed diffuse mild mesangial and endothelial proliferation with mesangial IgA deposits, and he received corticosteroids and cyclophosphamide for 1 year.

On examination, his weight and height were 50 kg and 170 cm respectively, with a body mass index of 17.3 kg/m^2^. Blood pressure was 120/93 mmHg. Other physical examination was unremarkable, tonsils were normal in appearance, the abdomen was soft and non-tender with normal bowel sounds, and he had no dependent edema. His initial laboratory workup was the following: creatinine, 96 μmol/L (40–135); estimated glomerular filtration rate, 101 ml/min/1.73 m^2^ (CKD-EPI formulae); albumin, 38.6 g/L (40–55); blood leucocytes, 5.56 × 10^9^/L (3.5–9.5); hemoglobin, 148 g/L (130–175); Other blood examinations were normal, including tests for antinuclear antibody, antineutrophil cytoplasmic antibodies, and complement C3 and C4. Urine microscopy showed 1765 RBC/high-power field with no casts, and 24-hour urine protein was 4.71 g.

**Figure 1:**
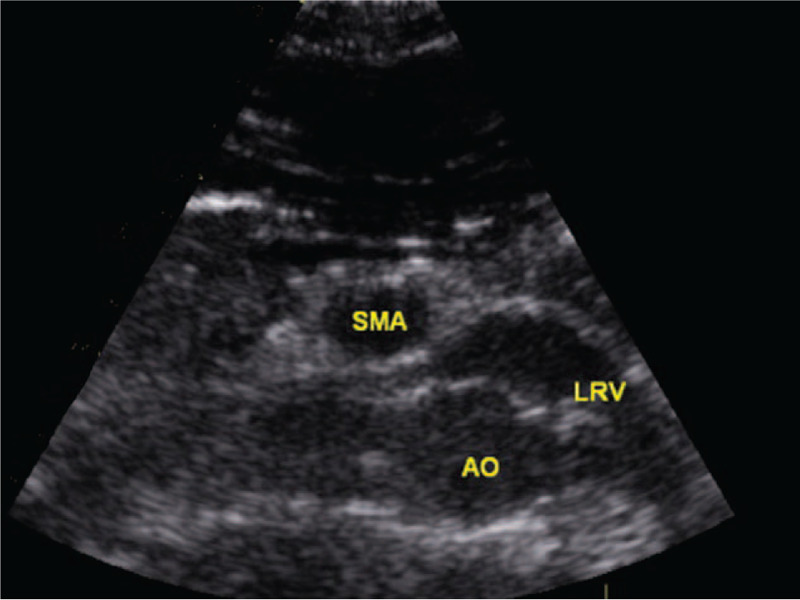
The Doppler ultrasonography revealed that left renal vein (LRV) that runs between the aorta (AO) and the superior mesenteric artery (SMA) is very narrow, which suggested nutcracker phenomenon.

**Figure 2:**
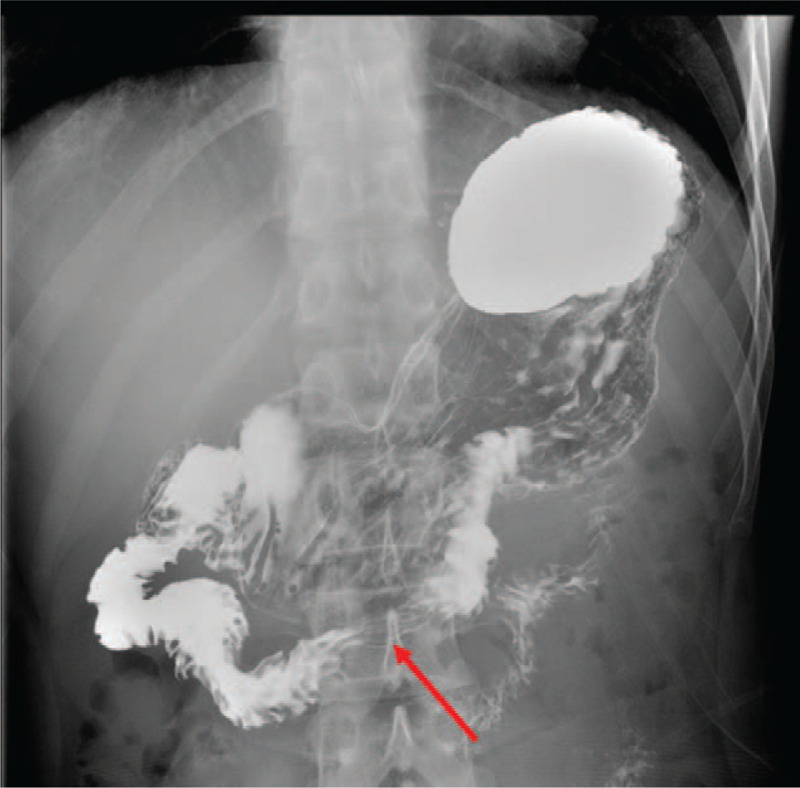
An upper gastrointestinal gastrografin study demonstrated compression of the third portion of the duodenum by the overlying superior mesenteric artery (red arrow). This suggests SMA syndrome.

Considering the persistent gross hematuria for more than 1 week with relatively normal renal function, Doppler ultrasonography was done, which revealed that LRV that runs between the aorta and the SMA is very narrow, and the anteroposterior diameter at the hilar portion divided by that at the aortomesenteric portion was 8.8 (1.06 cm/0.12 cm), and the ratio of LRV peak velocity (aortomesenteric portion to the hilar = 159 cm/sec/15 cm/sec) was 10.6 and the aortomesenteric angle was 10°. These findings were consistent with nutcracker syndrome (Fig. 1). After his admission, anorexia and abdominal distension with normal flatus and defecation were the most obvious discomforts, especially after eating, which needed parenteral nutrition supplementation. An upper gastrointestinal gastrografin study demonstrated compression of the third portion of the duodenum by the overlying superior mesenteric artery (Fig. 2). This suggests SMA syndrome.

**Figure 3:**
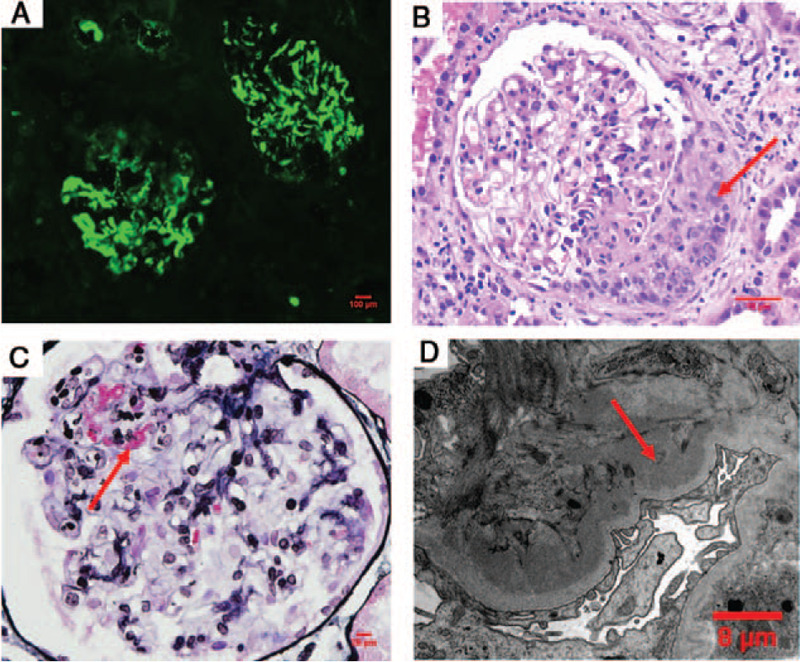
The pathological results of kidney biopsy. (A) Immunofluorescence showed IgA deposited in the mesangial areas. (B) Periodic Acid-Schiff (PAS) staining demonstrated cellular crescent (red arrow) with mild segmental mesangial proliferation and matrix expansion in the glomerulus. (C) Periodic Acid-Silver Methenamine (PASM)+Masson staining demonstrated segmental fibrinoid necrosis (red arrow) with segmental endothelial proliferation. (D) The electron microscopy displayed abundant deposits within the mesangial areas. (red arrow).

At this point, his serum creatinine increased to 186 μmol/L, and repeated percutaneous kidney biopsy was done at the right kidney, which showed IgAN with segmental glomerular necrotizing lesions in 24.3% of glomeruli, cellular crescents in 16.3% of glomeruli, interstitial fibrosis in 20% of the cortical area, and mild diffuse mesangial proliferation and matrix expansion (Fig. 3).

The decision was taken to initiate a pulse dose of methylprednisolone 500 mg daily for 3 days, followed by 1 mg/kg orally and mycophenolate mofetil 0.75 g twice a day. The patient's position was suggested to be maintained in the left lateral deviation position, prone position, or knee-chest posture. One month later, his abdomen symptoms such as anorexia and abdominal distension eased a lot with body weight increase of about 3 kg, serum creatinine decreased to 93 μmol/L, and urine microscopy showed 611 RBC/high-power field with 2.04 g urine protein per day. After 6 months of follow-up, his body weight increased to 57 kg, serum creatinine decreased to 63 μmol/L, and urine microscopy showed 75.5 RBC/high-power field with 0.3 g urine protein per day.

## 3 Discussion

In this case, the patient was diagnosed with IgAN for 7 years, and presented with episodes of macroscopic hematuria this time. Episodes of macroscopic hematuria is the typical clinical manifestation of IgAN, which usually follows mucosal infections, commonly in the upper respiratory tract or occasionally in the gastrointestinal tract.^[13,14]^ In such a scenario, gross hematuria usually resolves with control of associated infection.^[15]^ In this case, the patient had no obvious evidence of preceding infection.

Then combination of LRV entrapment was considered and confirmed by Doppler ultrasonography. LRV entrapment can be found at any age from childhood to the seventh decade, with prevalence peaking in patients who were in their second or third decade of life.^[16,17]^ Nutcracker can cause hematuria and proteinuria, even recurrent gross hematuria and persistent orthostatic proteinuria which might require surgical management.^[3]^ Cases of LRV entrapment combined with primary glomerular nephritis have been described, most of which were concurrent with IgA nephropathy and Henoch–Schoenlein syndrome. In a Japanese study, the prevalence of LRV entrapment in IgA nephropathy was 6.8%.^[18]^ Considering the relatively common combination of LRV entrapment and IgAN, a possible causal relationship between them has been raised,^[18,6]^ although not confirmed yet. Besides persistent gross hematuria, anorexia and abdominal distension with normal flatus and defecation were the most obvious discomforts of our patient, then SMA syndrome was diagnosed by an upper gastrointestinal gastrografin study. SMA syndrome is a rare cause of duodenal obstruction that presents with profound nausea and vomiting, abdominal distention, and post-prandial epigastric pain. SMA syndrome and nutcracker phenomenon have common features that result from narrowed aortomesenteric angle. However, it is very rare for both of them to occur simultaneously. The rare coexistence of SMA syndrome and nutcracker presenting in a patient with IgA nephropathy might indicate the relatively higher prevalence of vascular compression in IgA nephropathy, and further a supporter of a causal relationship between them. IgA induced gastrointestinal vasculitis should also be considered in this patient, however, the abdomen symptoms eased obviously when the patient's position was maintained in the left lateral deviation position, prone position, or knee-chest posture for 30 minutes after eating. Combined with the upper gastrointestinal gastrografin examination, we thought duodenal obstruction was the most probable diagnosis.

Then the patient developed acute renal failure with serum creatine increase from 96 μmol/L to 186 μmol/L. Macrohematuria associated acute renal failure is a widely known complication of IgAN, which was thought to be induced by hematuria itself through a tubular damage caused by intratubular erythrocytic casts. Duration of macrohematuria longer than 10 days, age > 50 year, decreased baseline estimated glomerular filtration rate, absence of previous episodes of macrohematuria, and the severity of tubular necrosis were risk factors for an incomplete recovery of kidney function. ^[19]^ For this patient, insufficient effective blood volume induced by the SMA syndrome might also be a contributor for the acute renal failure. Crescent nephropathy should be considered in IgAN patients with persistent macroscopic hematuria or deterioration of renal function.^[20]^ The combination of nutcracker syndrome and SMA syndrome made it difficult to identify the cause of acute renal failure in this patient, and a renal biopsy is necessary for a final diagnosis. Histopathological analysis of renal tissue confirmed the relatively severe renal injury. After immunosuppressive therapy combined with conservative therapy for SMA syndrome, abdomen symptoms eased a lot with a body weight increase, hematuria improved, and serum creatine decreased to the level even below the baseline.

## 4 Conclusion

Although the association between vascular compression and IgAN has not been elucidated yet, combination of nutcracker syndrome and SMA syndrome should be considered in patients with IgAN. The combination may increase the complexity of the disease, and renal biopsy should not be hesitated for differential diagnosis.

## Author contributions

**Investigation:** Bing Zhao, Liang Xu, Bing Liu, Qi Guo.

**Project administration:** Xiaowei Yang.

**Writing – original draft:** Chenghua Wang.

**Writing – review & editing:** Fengmei Wang, Rong Wang.
